# Neutrophil Functional Heterogeneity: Identification of Competitive Phagocytosis

**DOI:** 10.3389/fimmu.2017.01498

**Published:** 2017-11-09

**Authors:** Pien Hellebrekers, Falco Hietbrink, Nienke Vrisekoop, Luke P. H. Leenen, Leo Koenderman

**Affiliations:** ^1^Department of Respiratory Medicine, Laboratory of Translational Immunology, University Medical Center Utrecht, Utrecht, Netherlands; ^2^Department of Surgery, University Medical Center Utrecht, Utrecht, Netherlands

**Keywords:** granulocyte, bacteria, *Staphylococcus aureus*, innate immunity, PMN

## Abstract

**Introduction:**

Phagocytosis by neutrophils is a key process in the innate immune response against invading microorganisms. Despite reported heterogeneity in other neutrophils functions, little is known regarding differences in phagocytosis by individual cells. Therefore, we tested the hypothesis that heterogeneity is present in the neutrophil compartment in its potency to phagocytize bacteria.

**Methods:**

Phagocytosis assays were performed in suspension with isolated neutrophils and *Staphylococcus aureus* expressing different fluorescent proteins at MOIs between 1 and 10. Repetitive addition of bacteria with different fluorescent proteins and MOIs was used to compare the phagocytic capacity of *S. aureus*-green fluorescent protein (GFP)-positive and negative neutrophils and exclude randomness.

**Results:**

The percentage and mean fluorescence intensity (MFI) of *S. aureus*-GFP-positive neutrophils increased with higher MOIs. The increase in MFI was due to phagocytosis of multiple bacteria per neutrophil as was confirmed by confocal imaging. Sequential phagocytosis of GFP- and mCherry-expressing *S. aureus* showed a non-random process, as *S. aureus*-GFP-positive neutrophils preferentially phagocytized *S. aureus*-mCherry.

**Conclusion:**

All neutrophils were able to phagocytize *S. aureus*, but some were much more potent than others. Therefore, at physiologically relevant MOIs these potent phagocytizing neutrophils will outcompete the uptake of bacteria by less competent cells in a process we propose to name “competitive phagocytosis.”

## Introduction

Phagocytosis is an essential cellular mechanism involved in the killing of bacteria by the innate immune system (1). Engulfing the pathogen and providing a highly cytotoxic milieu inside the phagolysosome are the hallmarks in the killing mechanism (2). This prevents bacterial spreading within the host and provides protection against clinical manifest infections. Phagocytosis of microorganisms is known to be particularly executed by two cells from the innate immune system: macrophages and neutrophils (3). Apart from phagocytosis, a range of other mechanisms associated with bacterial killing have been identified. NET formation has been proposed as an additional mechanism for extracellular killing of microorganisms by neutrophils (4). In addition, neutrophils play an immunomodulatory role in the control in inflammation and cancer immunology (5). These different functionalities are likely mediated by a functional heterogeneity in the neutrophil compartment. Several studies have identified such functional compartmentalization (6). Recently, specific T cell suppressive neutrophil subsets have been described in acute inflammation and G-CSF-treated donors (7, 8). Several intra- and extracellular neutrophil markers have been proposed to define subsets in humans, yet none could be linked to functional heterogeneity in homeostasis (9).

Neutrophil phagocytosis capacity is known to be influenced by conditions such as age and disease. This is illustrated by the increased phagocytic activity in neutrophils in for example rheumatoid arthritis (10, 11). An *in vitro* assay with GM-CSF-treated human neutrophils showed enhanced phagocytosis in terms of the percentage of phagocytosing neutrophils as well as the phagocytotic activity expressed as number of bacteria per neutrophil compared to non-GM-CSF treated neutrophils (12). This suggests a reserve phagocytotic capacity of the neutrophil pool that requires priming to become active (13). On the other hand, in 1984 Stavemand Dahl observed that the distribution of the number of bacteria inside neutrophils when co-incubated in suspension deviated from the Poisson-distribution, which was to be expected in chance and equal capability. They hypothesized that the circulating neutrophils might be a heterogeneous population when it comes to phagocytosis/adherence of bacteria, but this old study lacks experimental data to exclude other possible explanations (14).

The differences in the interpretation of the above mentioned studies led us to hypothesize the presence of a spectrum in phagocytic capacity of individual cells in the human circulating neutrophil pool.

## Materials and Methods

### Human Volunteers and Ethical Aspects

Blood samples were provided by anonymous, healthy volunteers between the age of 18–65 years, male and female, after given written informed consent in accordance to de Declaration of Helsinki. All experiments were performed in accordance with the relevant guidelines and regulations. This study was approved by the University Medical Center Utrecht ethical review committee (METC).

### Neutrophil Isolation

Blood samples were collected in tubes with sodium heparin as anti-coagulant (Vacuette^®^ Greiner bio-one, Kremsmünster, Austria). For the isolation of the neutrophils, the blood was diluted in phosphate buffered saline and centrifuged over Ficoll-Paque (Pharmacia, Uppsala, Sweden) for 20 min at 760*g* at room temperature. The red blood cells in the pellet were lysed for 15–20 min in isotonic ice-cold NH_4_Cl solution. Cells were washed twice and resuspended in Hepes buffer (20 mM Hepes, 132 mM NaCl, 6 mM KCl, 1.2 mM KH_2_PO_4_, 1 mM MgSO_4_) supplemented with 5 mM glucose, 1 mM CaCl_2_, and 0.5% (w/v) human serum albumin. After washing, the neutrophils were resuspended in Hepes buffer and kept on ice until use. Absolute neutrophil counts were measured on an automatic hematology analyzer (CELL-DYN Emerald, Abbott, IL, USA). All preparations yielded >95% granulocytes with eosinophils as contaminating cells.

### Bacterial Growth and Colony Forming Units (CFU) Counting

Two strains of *Staphylococcus aureus* were used that were provided by the Laboratory of Microbiology at the UMC Utrecht: the MW2 strain expressing green fluorescent protein (GFP) (15) and the Reynolds CP5 (16) strain expressing mCherry both carrying chloramphenicol resistance. The strains were grown in Todd-Hewitt broth with chloramphenicol 10 µg/ml at 37°C and 180 rpm, until OD_600nm_ = 0.5. Hereafter, the suspension was centrifuged for 15 min at 3,500 rpm at room temperature and resuspended in Hepes buffer until OD_600nm_ = 0.5. The suspension was aliquoted in samples of 100 µl and frozen at −80°C until use. CFU were determined by dilution plating of the bacteria on Todd-Hewitt Agar plates.

### Phagocytosis Assays

#### Basic Phagocytosis Assay

Neutrophil suspensions with different neutrophil concentrations ranging between 1 × 10^6^ and 15 × 10^6^ neutrophils/ml were prepared in Hepes buffer in the presence or absence of 40% human serum. Bacteria were added to the neutrophil suspensions with different multiplicities of infection (MOI) ranging from 1 to 10. After addition of the bacteria the suspension was mixed gently and put in an incubator (Innova^®^ 44; New Brunswick Scientific, Edison, NJ, USA) at 37°C and 180 rpm. One sample of neutrophil–bacteria suspension was kept on ice to serve as a negative control. At different time points samples were taken from the neutrophil–bacteria suspension and fixed with 1% paraformaldehyde (PFA) for ≥15 min on ice.

#### Repeated Phagocytosis Assay

This assay consisted of two runs of the basic phagocytosis assay. A neutrophil suspension of 5 × 10^6^ neutrophils/ml was co-incubated with *S. aureus*-GFP with an MOI 1. After the first run, the neutrophils were sorted by forward- and side scatter to separate the neutrophils from the bacteria and by GFP expression to distinguish between *S. aureus*-GFP-positive and negative neutrophils. Cell sorting was performed on a FACSAria™ cell sorter (Becton Dickinson, Mountain View, CA, USA). The same phagocytosis assay was then repeated on the sorted *S. aureus*-GFP-negative neutrophils to show the distribution of the bacteria in this neutrophil population.

#### “Randomness” Assay

This assay consisted of two runs of the basic phagocytosis assay. A neutrophil suspension of 5 × 10^6^ neutrophils/ml with 40% human serum was used for all randomness assays. In the first round *S. aureus*-GFP was added to the neutrophil suspension with either a MOI 1 or 10. After 40 min, a second round of bacteria was added to the same sample, but now *S. aureus*-mCherry with a MOI 1 or 2. As a control a reverse assay was also performed with the *S. aureus-*mCherry in the first round and -GFP in the second round. First rounds without bacteria were conducted as a control. Samples were taken at different time points: before round 1 (baseline), before the second round at 40 min and at the end of the experiment at 80 min. The cells were fixed immediately with 1% PFA and put on ice for at least 10 min. Combinations and their purpose are shown in Table 1.

**Table 1:** Phagocytosis assays to assess randomness and feed-forward mechansims.

First round	Second round	Aim
No bacteria	MOI 1/2	Serve as a control for phagocytizing capability after 40 min at 37°C
No bacteria	MOI 10	

MOI 1	MOI 1/2	Observe and compare distributions of first and second round bacteria
MOI 1	MOI 10	

MOI 10	MOI 1/2	Observe the effect of activation of all neutrophils on second round phagocytosis
MOI 10	MOI 10	

*Staphylococcus aureus-green fluorescent protein was mostly used in the first round and -mCherry in the second*.

#### Feed-Forward Assay

This assay is similar to the “randomness” assay described above, but differs in the fact that the first round is executed with *S. aureus*-GFP with MOI 10. Under these conditions almost all (>95%) neutrophils phagocytized at least one bacterium in the first round and a possible feed-forward mechanism could be initiated. The distribution pattern of the *S. aureus*-mCherry (MOI1 and 2) in the second round can then distinguish between feed-forward and an inherent propensity for phagocytosis. See Table 1 for an overview of combinations of MOI in the first and second round and their objectives.

### Flow Cytometry Analysis

The fixed cells were analyzed using a BD LSRFortessa™ cell analyzer (Becton Dickinson, Mountain View, CA, USA). Neutrophils were identified according to their specific forward-/side- scatter patterns. Eosinophils were excluded based on their auto-fluorescence in the FL-1 channel. Data from the experiments are depicted as percentages of GFP or mCherry positive neutrophils and mean fluorescence intensity (MFI) of at least 10,000 events.

Multicolor analysis was performed to analyze marker expression and to possibly differentiate between cells with high and low phagocytosing capacity. The neutrophils were stained after phagocytosis for 30 min on ice in the dark before fixation with 1% PFA. Antibodies used for this purpose were CD16-Krome Orange (3G8), CD62L-ECD (DREG56), and CD11b-APC-AF750 (Bear1) from Beckman Coulter (Pasadena, CA, USA). CD16-V500 (3G8), CD32-APC (FLI8.26), and the Cytofix/Cytoperm^TM^ from BD (San Jose, CA, USA). CD11c-PerCP-Cy5.5 (BU15), CD49d-PeCy7 (9F10), CD66b-PerCP-Cy5.5 (G10F5), and CD64-Pacific Blue (10.1) from Biolegend (San Diego, CA, USA). CD35-PE (AR2), streptavidin-AF647 and EZ-link Sulfo-NHS-LC-Biotin from Thermo Fisher Scientific (Waltham, MA, USA). CD88-PE (P12/1) and OLFM-4 (N-20) from Santa Cruz (Dallas, TX, USA), CD182-APC (48311) from R&D systems (Minneapolis, MN, USA), flow cytometry data were analyzed with FlowJo^®^ v10 software (FlowJo, LLC, Ashland, OR, USA).

### Confocal Imaging

For confocal imaging neutrophils were stained after the basic phagocytosis (*S. aureus*-GFP) experiment with CD16-V500 (clone 3G8, Becton Dickinson, Mountain View, CA, USA) for 20 min on ice in the dark. Hereafter, the neutrophils were fixed using PFA 1% as before mentioned. Fixed cells were imaged using Zeiss LSM confocal microscope (Carl Zeiss Microscopy GmbH, Lena, Germany). Extracellular CD16-V500 staining was used to determine the cell borders of the neutrophil. Z-stacks were performed to determine the location of the *S. aureus*-GFP and measure the number of bacteria inside the cell. Image analysis was performed using Zen2009 (Zeiss) and ImageJ software (Fiji, Madison, WI, USA).

### Statistical Analysis

Data are expressed as means ± SEM. Normal distribution of the data was tested using D’Agostino and Pearson normality test. Comparisons between conditions were made with the Wilcoxon test, or one-way ANOVA in case of multiple conditions. The Fisher LSD test was used for multiple comparisons, *P*-values reported are not adjusted for multiple comparisons. Results were regarded as significant when *P* < 0.05.

### Data Availability

The datasets generated during and/or analyzed during the current study are available from the corresponding author on reasonable request.

## Results

### Active Phagocytosis in Only Part of the Neutrophil Population

**Figure 1:**
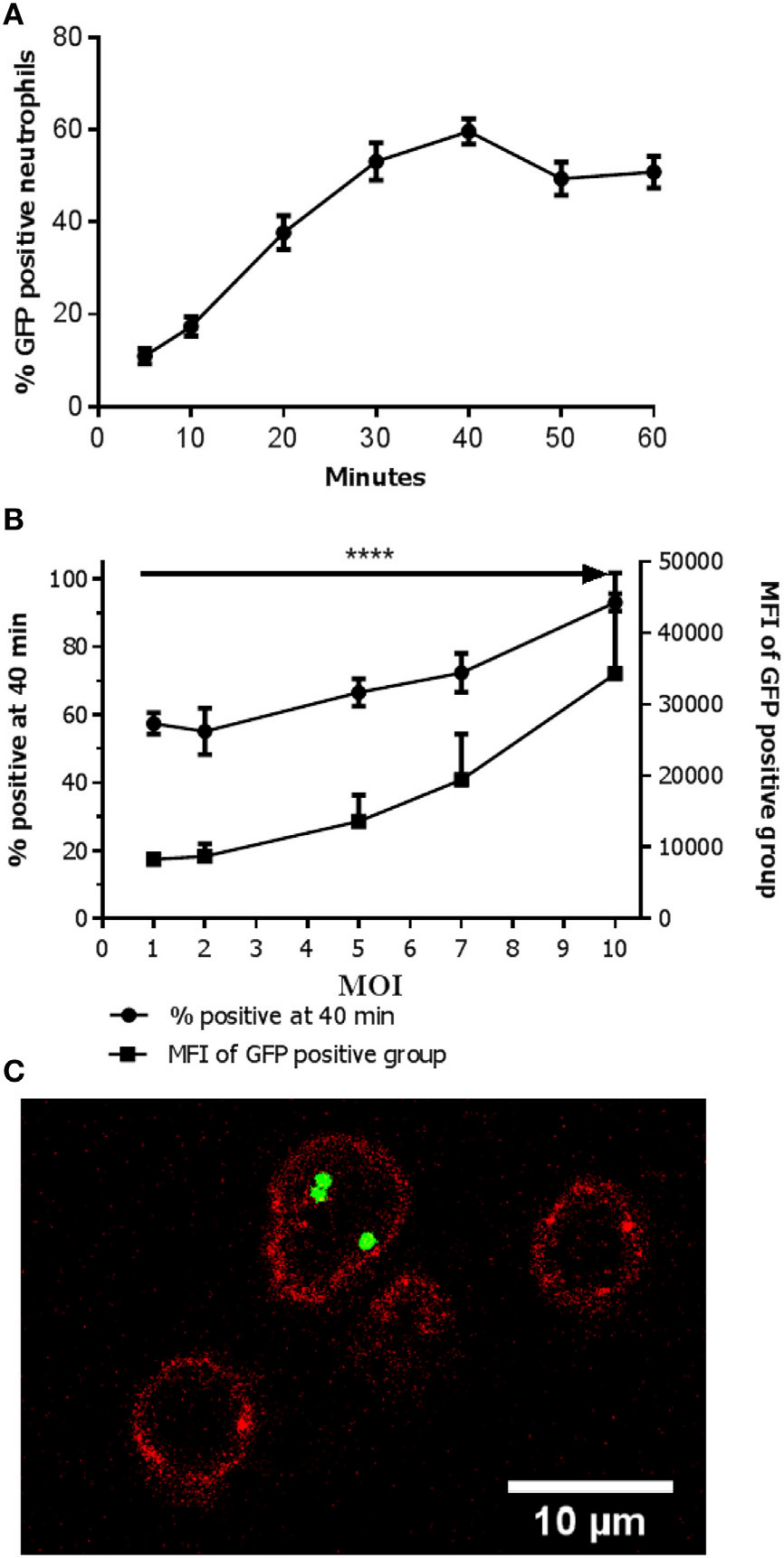
Active phagocytosis in only part of the neutrophils population. **(A)** Percentage of *Staphylococcus aureus*- green fluorescent protein (GFP)-positive neutrophils over time. The percentage of positive neutrophils is depicted when incubated with an MOI 1. A plateau is reached at 40 min. Data are presented as mean ± SEM [*n* = 17 (5 min), 15 (10 min), 17 (20 min), 13 (30 min), 29 (40 min), 10 (50 min), 12 (60 min)]. **(B)** Percentage of *S. aureus*-GFP-positive neutrophils with increasing MOI. The percentage of positive neutrophils in conditions with increasing MOI is depicted. An increase in the percentage of *S. aureus-*GFP-positive neutrophils as well as concurrent increase in mean fluorescence intensity (MFI) is observed (****P < 0.0001). Data are presented as mean ± SEM [*n* = 8 (MOI 1), 5 (MOI 2), 6 (MOI 5), 5 (MOI 7), 8 (MOI 10)]. Data were analyzed with one-way ANOVA. **(C)** Confocal image showing a neutrophil (cell membrane is visualized in red by staining of CD16-V500) with multiple intracellular bacteria (green, GFP), and two empty neutrophils.

Neutrophils that were incubated with bacteria but in the absence of human serum showed nearly no phagocytosis (results not shown) as has also been described before (17, 18). In suspension with 40% human serum in a shaking incubator in the presence of GFP positive bacteria (MW2) at a multiplicity of infection (MOI) of 1, the percentage of GFP positive neutrophils reached a plateau after 40 min (Figure 1A). Therefore, the time point 40 min was chosen as the endpoint in the remainder of the experiments. Suspensions with increasing MOI showed an elevation in the percentage of GFP positive neutrophils (Figure 1B). Notably, the MFI of the GFP positive fraction of the neutrophils increased concurrently as the MOI increased, but negative neutrophils still persisted. This suggested active phagocytosis of multiple targets in some neutrophils and no phagocytosis in others. This was confirmed with confocal microscopy as shown in Figure 1C. Only low numbers of adhering bacteria were seen and thus bacterial adhesion to the outside of the plasma membrane seems to contribute only minimally to the phenomenon seen in flow cytometry. For further experiments MOIs of 1 or 10 were chosen to mimic *in vivo* situations of bacterial control and bacteremia, respectively.

### Phagocytic Capability Differs within the Neutrophil Population

Upon increasing MOIs a rise in MFI was seen in the neutrophil population that phagocytized the bacteria (see Figure 1B). Despite the increase in MFI (i.e., phagocytosis of more bacteria) a clear negative population remained, caused by phagocytosis of more bacteria by some neutrophils versus no phagocytosis in others. However, with even higher MOIs virtually all neutrophils phagocytized one or more bacteria (see Figure 1B). These findings suggest that although all cells are capable to phagocytize bacteria there are neutrophils more actively phagocytizing bacteria than others. However, when sufficient numbers of bacteria are present (MOI of 10) nearly all (>97%) of the neutrophils are capable of phagocytosis.

**Figure 2:**
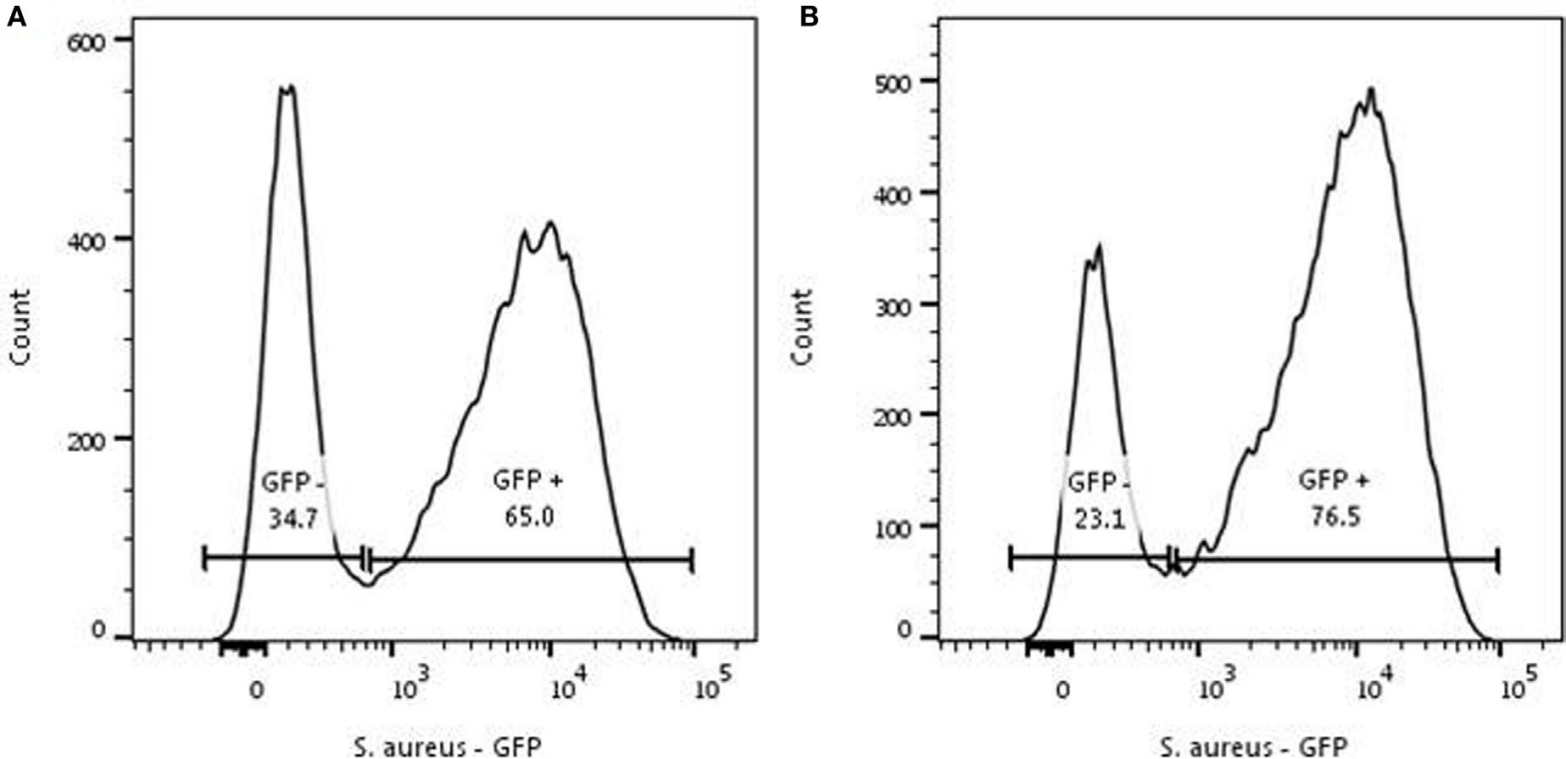
Repeated phagocytosis experiment. Representative experiment for two individual experiments. **(A)** Phagocytosis of *Staphylococcus aureus-* green fluorescent protein (GFP) by neutrophils (5 × 10^6^/ml) was initiated with a MOI of 1 for 40 min. The GFP-negative cells were sorted and the sorted cells underwent the same procedure as the cells depicted in panel **(A)**. **(B)** Sorted GFP-negative neutrophils were mixed with *S. aureus-*GFP for 40 min and analyzed again. A comparable distribution of *S. aureus*-GFP is seen in the formerly GFP-negative population.

A repeated phagocytosis assay was performed to determine the capacity of phagocytosis by the initial negative (non-phagocytosing population) group 40 min after incubation with an MOI 1. Here, the negative population was FACS-sorted and again incubated with *S. aureus* at a MOI of 1. This showed a comparable distribution of GFP-positive and negative neutrophils in the repeated and the initial phagocytosis experiment (Figure 2).

Both the basic phagocytosis experiment and the repeated phagocytosis assay with sorted GFP negative neutrophils demonstrated that all cells have a phagocytosis capability, but suggest a spectrum in the capacity of individual cells for phagocytosis of *S. aureus*.

### A Spectrum in the Capacity of the Neutrophils to Phagocytize Bacteria

**Figure 3:**
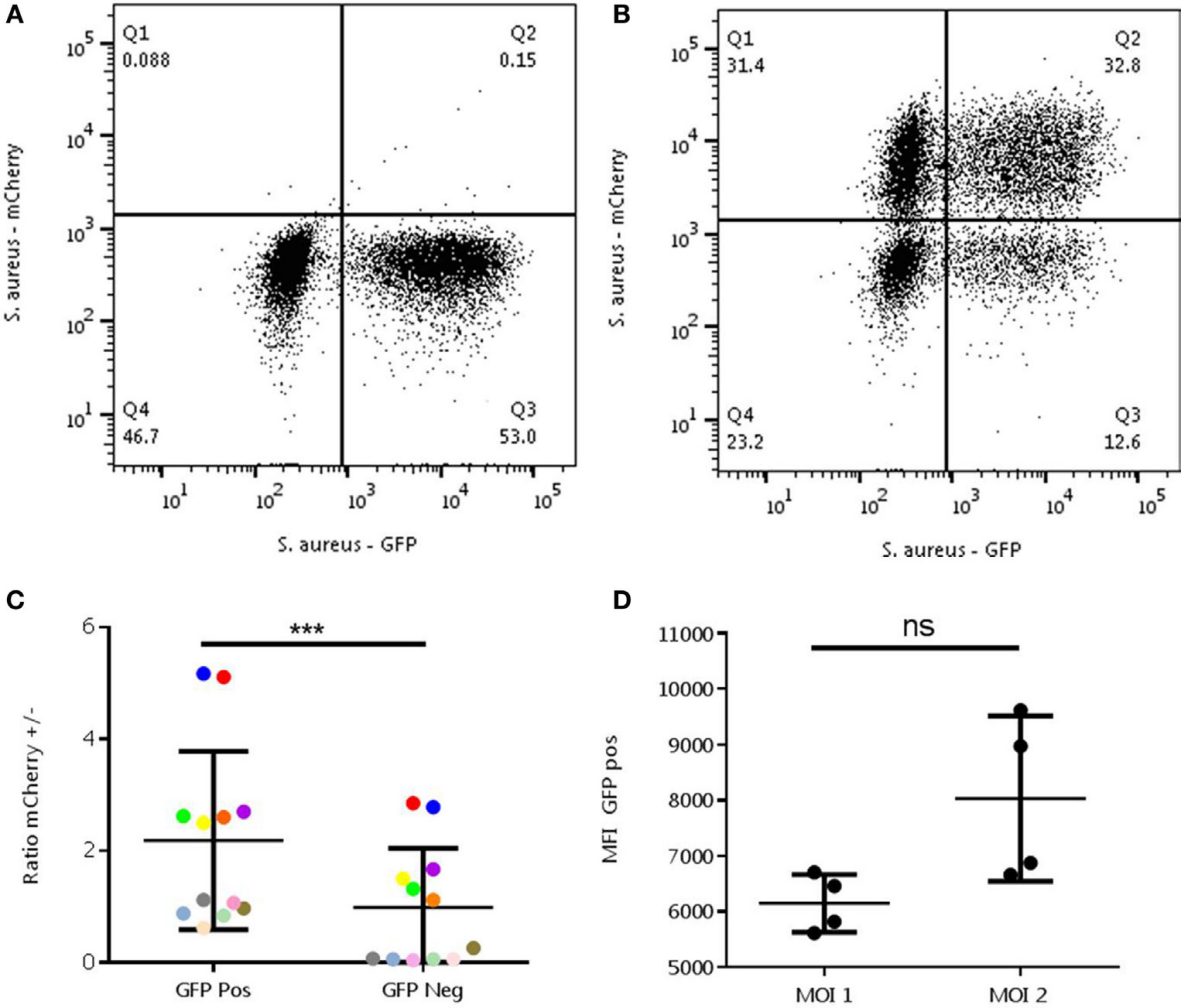
Analysis of randomness in the interaction between *Staphylococcus aureus-* green fluorescent protein (GFP) and neutrophils. The experiment was performed with a MOI of 1 *S. aureus-*GFP in the first round **(A)** and with -mCherry in the second round **(B)**. A representative experiment is shown. Ratios in the mCherry positive (/+) and negative cells (/−) were determined in the GFP-positive (+/) and negative (−/) **(C)** and MOI 1 and 2 **(D)** cells. **(C)** A relative enrichment of +/+ and −/− neutrophils is seen compared to the +/− and −/+ cells, which is not to be expected in a random process (***P = 0.0005). Differently colored dots represents results from the same experiments, different experiments can be executed with different MOIs. **(D)** An increase in mean fluorescence intensity can be observed in the mCherry positive group of the MOI 2 condition compared to a MOI 1 (ns, *P* = 0.125). Data were analyzed with the Wilcoxon test.

The abovementioned experiments might be interpreted that random chance for the interaction between cells and bacteria plays an important role. To rule out such a stochastic mechanism, we designed an experiment that could discriminate between randomness and pre-determined differences in phagocytic capacity. In the first round, the neutrophils were incubated with *S. aureus*-GFP with an MOI 1. In this phase again a clear negative population is seen (see Figure 3A). If phagocytosis is indeed a random process, a second round with alternatively colored bacteria would be expected to result in a random distribution over the cells. Therefore, a second round of infection was executed with *S. aureus*-mCherry with an MOI 1 or 2. An MOI 2 was added to be sure to provide enough bacteria per neutrophil for sufficient phagocytosis for all neutrophils, and simultaneously see the presence of negative cells and an increase in MFI in the positive neutrophils. A representative experiment is shown in Figure 3A, first round, and Figure 3B, second round.

Ratios of mCherry positive and negative were determined and MFI was measured. Ratios of mCherry positive and negative neutrophils were not equally distributed across the GFP-positive and negative populations, which was expected in a random process. In fact, comparisons of the ratios showed a significant difference with higher mCherry positive neutrophils present in the GFP-positive group (*P* < 0.0005). Also, the MFI of GFP (a measure of bacterial number) was higher in the mCherry positive group when incubated with an MOI 2 compared to MOI 1(*P* = 0.125). This demonstrates that phagocytosis of *S. aureus*-mCherry was higher in the cells that already have phagocytized the *S. aureus*-GFP in the first round (Figures 3C,D).

The data indicate that phagocytosis by neutrophils is not a random process. Next, the hypothesis was tested whether the underlying mechanism causing the distribution of phagocytizing and non-phagocytizing neutrophils could either be a feed-forward phenomenon (once a bacteria is phagocytized the phagocytosis of the next bacterium is facilitated) or that there is an intrinsic heterogeneity in the capacity of the neutrophils to phagocytize bacteria. To distinguish between these two hypotheses, the double phagocytosis experiment was repeated with an MOI 10 in the first round and addition of alternatively colored bacteria with an MOI of 1 in the second round. Under these conditions all neutrophils phagocytized at least one bacterium in the first round. If a feed-forward phenomenon would be present, we expected a more random distribution of mCherry positive neutrophils in the second round. Activation of all neutrophils through a feed-forward mechanism with equal chances would result in a more Gaussian distribution of the *S. aureus*-mCherry rather than a distribution over two distinct populations with an increase in MFI.

**Figure 4:**
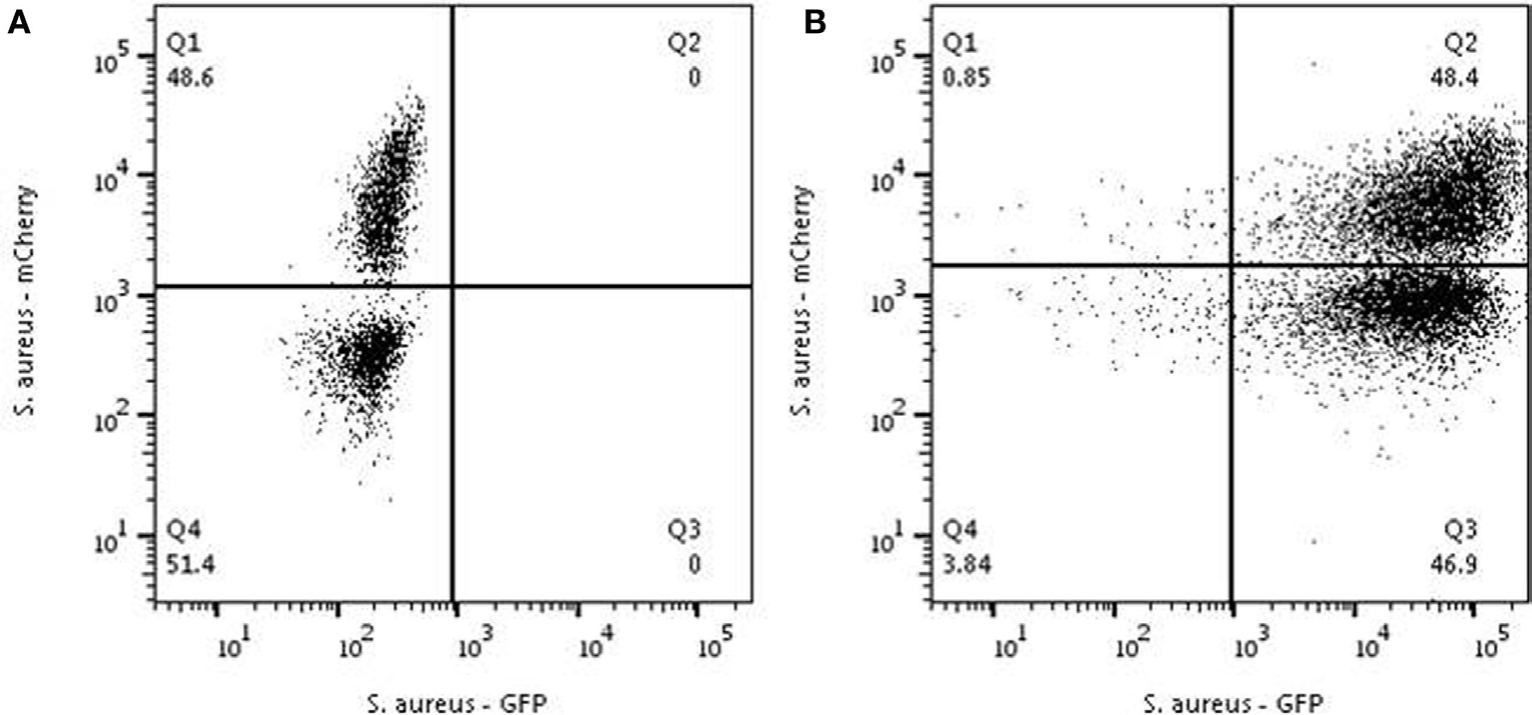
Analysis to test the feed-forward hypothesis. Neutrophils were first mixed with *Staphylococcus aureus -*Cherry with an MOI of 1 and a clear distribution is seen into positive and negative cells **(A)**. **(B)** The same experiment was performed in cells from the same donor preincubated with *S. aureus-*GFP at an MOI of 10. All neutrophils were characterized in the first round by 100% phagocytosis all neutrophils become GFP-positive[panel **(B)**]. Adding *S. aureus-*mCherry with a MOI of 1 to these GFP-positive neutrophils resulted the same distribution pattern that was observed before adding *S. aureus-*GFP [see panel **(A)**]. Depicted are representative samples for three independent experiments.

Phagocytosis of all neutrophils in the first round did not alter the distribution of *S. aureus-*mCherry in the second round (Figure 4). This argued against a feed-forward phenomenon and supports the hypothesis for an intrinsic difference in the capacity of phagocytosis between individual neutrophils.

To evaluate whether the neutrophils with high phagocytosis capacity could be distinguished from the neutrophils with low capacity, a multicolor flow cytometry analysis was performed before and after one run of the basic phagocytosis assay. Differences in extracellular marker expression and OLFM-4 positivity between the *S. aureus*-GFP-positive and negative neutrophils were analyzed. Only minor differences in marker expression were found between the positive and negative neutrophils. No difference in the expression of OLFM-4 was detected. (Results not shown.)

## Discussion

This is to our knowledge the first study that describes a spectrum in phagocytic capacity of cells in the human circulating peripheral neutrophil pool under healthy conditions. The phagocytosis experiments clearly show an increase in MFI of neutrophils phagocytosing multiple *S. aureus*-GFP compared with the relatively large GFP-negative neutrophil population. This strongly suggests a preference of some neutrophils over others to phagocytize better their targets. Essentially, three explanations could underlie this phagocytic behavior of neutrophils; (1) An intrinsic functional difference between cells; (2) randomness in the interaction between cells and bacteria that might be affected by the design of the experiment; and (3) a feed-forward phenomenon by which the phagocytosis of the first bacterium facilitates this process for the next bacteria. To exclude the latter two options we designed an experiment to exclude randomness in which we co-incubated the neutrophils consecutively with two differently colored *S. aureus* expressing different fluorescent proteins. These data clearly demonstrate a spectrum of the cells with respect to their phagocytic capacity.

Neutrophils with a high capacity in a first round of phagocytosis are characterized by a similarly high capacity for additional phagocytosis with alternatively colored bacteria (see Figure 3). This could be explained by a feed-forward phenomenon (i.e., phagocytosis of a bacterium facilitates the uptake of the next organism) often seen in immunological reactions. However, this was ruled out by the result of the experiment, where all neutrophils were allowed to phagocytize *S. aureus*-GFP first (incubation with an MOI of 10) followed by addition of *S. aureus*-mCherry at an MOI 1. A feed-forward mechanism would have led to a random distribution of *S. aureus*-mCherry in the neutrophils with *S. aureus*-GFP present in their phagolysomes. In marked contrast, our data showed that the distribution of highly phagocytic neutrophils is independent of previous phagocytosis of bacteria. This is in line with the hypothesis that neutrophils with a high capacity to phagocytize bacteria do this because of an intrinsic phenotype rather than an induced phenotype. It is important to emphasize that all neutrophils appear capable of phagocytosis, when providing sufficient amounts of bacteria (see Figure 1B). Also in experiments where non-phagocytosing neutrophils were sorted it was shown that these cells could phagocytize bacteria (see Figure 2). These data provide evidence for a spectrum in the phagocytic function in neutrophils in blood of normal individuals and that there is a competition in phagocytosis between highly phagocytic cells and lowly phagocytic cells. We propose to name this process competitive phagocytosis.

This heterogeneity in phagocytic capacity of different neutrophils prompted us to evaluate whether we could phenotype these cells, such that it would be possible to differentiate between these cells with the use of surface marker expression. Multicolor analysis with twelve common neutrophil markers did not result in the identification of phenotypes associated with phagocytic function (results not shown). The lack of distinctive markers before phagocytosis is in line with the consensus that neutrophils belong to a homogenous population of cells in the blood of healthy individuals when studied in the context of expression of cell surface markers. There were only slight differences in expression of these markers on all neutrophils when incubated with bacteria indicating transcellular activation as a consequence of phagocytosis. None of the markers identified potent phagocytosing cells before adding of the bacteria.

We investigated the OLFM-4 expression as a marker for neutrophils with a high capacity of phagocytosis. No differences in distribution of GFP-positive and negative neutrophils between OLFM-4 positive and negative neutrophils were seen (results not shown). Therefore, OLFM-4 is not a marker for phagocytosis prone neutrophils. Clemmensen et al. reported OLFM-4 expression to define two subsets of neutrophils without a clear functional consequence for this expression on human neutrophils (19). Some immunological functions have been attributed the presence or absence of OLFM-4 subsets in the mouse. For example, enhanced bactericidal capacity was detected against *S. aureus* leading to resistance for sepsis in mice (20, 21). However, we could not link OLFM-4 expression to the intrinsic capacity of neutrophils to phagocytosis.

It is well known that the phagocytic capacity of neutrophils is not a “fixed” cellular characteristic. Phagocytosis can be influenced by both external, e.g., temperature, medication, injuries (22–24), and internal factors, e.g., age, sepsis, auto-immune disease (25–27). Also, *ex vivo* manipulation can alter the function of a single sample of neutrophils with regard to phagocytosis (14, 28). However, the main objective of this study was to observe heterogeneity in phagocytosis capacity of single neutrophils within one individual in homeostasis. Although, the aforementioned factors are very relevant for the understanding of neutrophil functionality in general, they were beyond the scope of this study.

As also stated in the introduction, variation in phagocytosis within the neutrophil pool of a single human has been observed before (12, 29). This heterogeneity has never been studied in terms of a possible underlying mechanism. By not ruling out randomness, feed-forward mechanisms and “non-fit” cells, a concept of competitive phagocytosis within the neutrophil pool could not be tested in these earlier studies. Previously, a spectrum in the maximum capacity of neutrophils for phagocytosis of bacteria has also been shown (30). Clawson and Repine studied phagocytosis capacity using ratios of up to 400 *S. aureus* per neutrophil, resulting in an average of 47 bacteria per neutrophil (30). Since bacterial numbers used in our study are far from saturating the maximum phagocytosis capacity, this is of no significance for the interpretation of our results.

Our FACS assay was based on the use of live GFP/mCherry expressing *S. aureus*. This assay cannot distinguish between phagocytized and adhering bacteria. We chose to make no discrimination between the two mechanisms, because adherence without phagocytosis was almost absent in neutrophils from healthy humans and is considered an “early phase of phagocytosis” (31). Furthermore, there was an absence of adhering bacteria in our confocal microscopy images and during real-time imaging, which makes it unlikely that the discrimination between the two processes would have led to a difference in interpretation of our results.

Although the sole use of *S. aureus* was sufficient to accomplish proof-of-principle, additional studies should be performed to confirm this spectrum in neutrophils for phagocytosis of other bacteria. An early study of Dijkmans et al., showed altered phagocytosis of *Escherichia coli* by neutrophils when simultaneously exposed to *S. aureus* (32). They mainly attributed this to competitive opsonization, but multiple mechanisms or intrinsic differences may have played a role. More studies on different bacteria and bacteria combinations may provide further insight into the spectrum and putative specificity of phagocytosis capability of human neutrophils.

To our knowledge, this is the first description of competitive phagocytosis in human neutrophils. One can only speculate on the evolutionary advantage of this process. It might be beneficial for the neutrophil compartment to be able to fine-tune antibacterial responses by a limited number of neutrophils when faced with an acute infection, because collateral damage by hyperactivation of the complete neutrophil compartment can lead to multiple-organ dysfunction syndrome and death (33). Future studies should also focus on the importance of cells exhibiting a low capacity to phagocytize bacteria for their capability of other neutrophil functions such immune regulation.

## Ethics Statement

Blood samples were provided by healthy volunteers after given written informed consent in accordance to de Declaration of Helsinki. All experiments were performed in accordance with the relevant guidelines and regulations. This study was approved by the University Medical Center Utrecht ethical review committee (METC).

## Author Contributions

PH was responsible for setting up and execution of the experiments, interpretation of data, and drafting of the manuscript. LK was responsible for setting up the experiments, interpretation of data, and critically reviewing the manuscript and approved of the final version. NV, FH, and LL were responsible for interpretation of the data and critically reviewing the manuscript and approved of the final version.

## Conflict of Interest Statement

The authors declare that the research was conducted in the absence of any commercial or financial relationships that could be construed as a potential conflict of interest.
